# Recognition of 3′-end L1, Alu, processed pseudogenes, and mRNA stem-loops in the human genome using sequence-based and structure-based machine-learning models

**DOI:** 10.1038/s41598-019-43403-3

**Published:** 2019-05-10

**Authors:** Alexander Shein, Anton Zaikin, Maria Poptsova

**Affiliations:** Laboratory of Bioinformatics, Big Data and Information Retrieval School, Faculty of Computer Science, National Research University Higher School of Economics, Moscow, Russia

**Keywords:** Computational models, Data processing

## Abstract

The role of 3′-end stem-loops in retrotransposition was experimentally demonstrated for transposons of various species, where LINE-SINE retrotransposons share the same 3′-end sequences, containing a stem-loop. We have discovered that 62–68% of processed pseduogenes and mRNAs also have 3′-end stem-loops. We investigated the properties of 3′-end stem-loops of human L1s, Alus, processed pseudogenes and mRNAs that do not share the same sequences, but all have 3′-end stem-loops. We have built sequence-based and structure-based machine-learning models that are able to recognize 3′-end L1, Alu, processed pseudogene and mRNA stem-loops with high performance. The sequence-based models use only sequence information and capture compositional bias in 3′-ends. The structure-based models consider physical, chemical and geometrical properties of dinucleotides composing a stem and position-specific nucleotide content of a loop and a bulge. The most important parameters include shift, tilt, rise, and hydrophilicity. The obtained results clearly point to the existence of structural constrains for 3′-end stem-loops of L1 and Alu, which are probably important for transposition, and reveal the potential of mRNAs to be recognized by the L1 machinery. The proposed approach is applicable to a broader task of recognizing RNA (DNA) secondary structures. The constructed models are freely available at github (https://github.com/AlexShein/transposons/).

## Introduction

Transposons are pieces of DNA, which are able to multiply and move inside a genome. Transposons have been found in all eukaryotes and usually occupy a considerable part of any genome (46% of a human, 37% of a mouse, 10% of a fruit fly, and 85% of a corn genome)^1,2^. For a long time, transposons were considered as junk DNA, which do not have any function. However, after discovering haemophilia that was caused by transposon insertion into the gene of a coagulation factor^3^, the pathogenic role of transposons became evident. Nowadays, 96 human diseases caused by a transposon insertion have been discovered^4^. Many studies were devoted to the role of transposons in cancer formation^1,5^. Transposons made an important contribution to the variability of genomes of many organs, for example, the brain and the immune system^6^. Transposons started to be considered as a “tool” of evolution, because they cause large-scale genome re-arrangements (for example, recombination between two non-allelic elements in two different chromosomes) as well as minor genome changes (duplications, inversions, deletions)^7^. Transposons can influence their own expression as well as the expression of the nearby genes^8^.

For a transposition, long interspersed nuclear elements (LINEs) use their own molecular machinery encoded in the LINE sequence. This machinery is employed for copying the sequence and for its subsequent insertion into the genome. Short interspersed nuclear elements (SINEs) are parasites, they do not encode proteins, and they require the machinery of LINE to perform transposition. How LINE proteins recognize their own RNA and SINE RNA remains unclear^8^. For several species it was experimentally shown that the LINE protein recognizes a secondary structure, such as a stem-loop, at the 3′-end of a transposon RNA. Moreover, it was shown that in some organisms, LINEs and SINEs have identical 3′-end sequences containing a stem-loop structure, which is essential for transposon RNA recognition by transposon proteins^9–11^. In case when transposons do not have an identical 3′-end, it is thought that a poly-A tail serves as a recognition element of SINEs and LINEs. However, almost all mRNAs have poly-A tails and this does not explain the specific recognition of transposon sequences by the LINE-encoded proteins. We discovered a conserved secondary structure at the 3′-end of human L1 (LINE) and Alu (SINE) transposons^12^, as well as in different species across the tree of life (unpublished results). Despite the absence of similarity at the level of sequences, the conserved position of this structure suggests its functionality.

Solution 3D-structures of stem-loops for LINE transposons in eel and zebrafish revealed some characteristic structural stem-loop properties that, together with mutagenesis analysis, showed which nucleotide positions are vitally important for retrotransposition. Thus, the solution structure of RNA stem-loop from 3′UTR of eel LINE transposons UnaL2 determined the specific structure of the loop GGAUA^13^. The fourth uridine appeared to be exposed to solvent, but mutation of this residue did not affect retrotransposition. The third and fifth adenosine residues are stacked, and their mutations decreased the activity. Mutation of the second guanosine diminished retrotransposition activity. The authors hypothesized that the second guanosine in the loop is directly recognized by UnaL2 reverse transcriptase (RT).

Another study showed that an internal loop is also required for retrotransposition^14^ because its deletion completely blocked the activity. With the help of molecular dynamic simulations the authors showed that an internal loop acts as a hinge allowing stem-loop to have conformational flexibility. Mutational analysis showed that this flexibility is somehow required for transposition.

Structures of stem-loops of two transposons from zebrafish ZfL2-1 and ZfL2-2 were examined^9^. Stem-loop in one transposon ZfL2-1 has an insertion in the form of the second stem-loop at the place of the 4^th^ residue in the loop in the ZfL2-2. Thus, the loop of ZfL2-2 became an internal loop in ZfL2-1. The experiments with mutual loop replacements with the stem region showed that retrotransposition was almost reduced to zero in both cases. This points to a specificity in loop recognition. An importance of loop and internal loop in stem-loop recognition for zebrafish ZfL2-1 and ZfL2-2 transposons, and specifically of certain residues, was also demonstrated in this study^15^.

Here, we investigated the structural stem-loop properties of human L1 and Alu transposons with the help of two types of machine-learning models: one is built using information about sequence composition and the other using structural, physical, and chemical properties of ribonucleic acid molecules. Taking into account the importance of position-specific nucleotides in loops and in bulges, we treated structural characteristics of stems and loops separately. For stem characteristics we used dinucleotide properties available from DiProDB database^16^: Gibbs energy, enthalpy, entropy, hydrophilicity, and geometrical structural parameters of RNA molecule: shift, slide, rise, tilt, roll, and twist. For loops and bulges we fixed positions in order to capture position-specific preferences for nucleotides in certain positions.

Dinucleotide characteristics were already used in machine-learning models to predict specific genomic sites. For example, protein binding was predicted based on DNA dinucleotide properties^17,18^. Also, using dinucleotide physical and geometrical properties it was possible to build machine-learning models, which recognize recombination sites^19^, splice-sites^20^, regulatory small RNA originated from transposon sequences^21^, DNA-editing sites^18^ with high accuracy. Sequence-based models using frequencies of di- and tri-nucleotides were successfully applied to predict quadruplexes^22^.

The goal of the present study is to construct machine learning models that can be trained to separate stem-loop structures at the 3′-end of human L1 and Alu transposons from stem-loops from shuffled sequences, and also to test constructed models on mRNAs and processed pseudogenes. We built two types of models – sequence-based models, which use information on nucleotide composition, and structure-based models that consider physical, chemical and geometrical properties of a stem and position-specific nucleotides of a loop and a bulge. Both models were able to recognize L1 and Alu 3′-end stem-loops with high performance (97–99% AUC), and 3′-end stem-loops of L1 and mRNAs or processed pseudogenes with moderate performance. Structure-based models helped to reveal properties that are most important for model prediction power, which appeared to be shift, rise, and hydrophilicity, as well as stem positions such as the dinucleotides adjacent to the loop and the bulge. We discuss the biological significance of top influential characteristics for RNA-protein recognition. The constructed models can be used for *de novo* discovery of retrotransposon-related stem-loop structures.

## Results

### Recognition of 3′-ends and 3′-end stem-loops of L1s and Alus

We took full-length sequences of 6,622 L1 elements and 12,431 Alu elements (see Methods) from two evolutionary studies identifying active families of transposons in the human genome^23,24^. We annotated all transposons with stem-loop structures (see Methods) and composed sets of stem-loops located at the 3′-end of transposons. Additionally, we composed sets of stem-loops from 5′-end regions of both Alu and L1 transposons and created shuffled sequences as an alternative class (see Methods).

**Figure 1:**
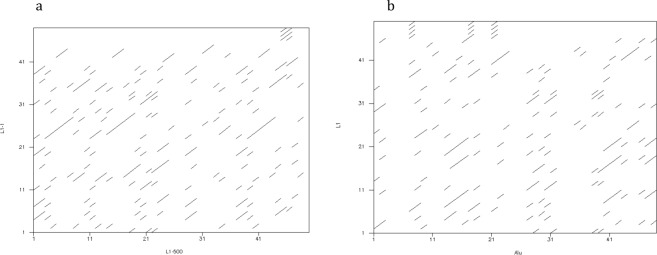
Dotplots of 3′-end 50 bp L1 and Alu sequences. (**a**) Two sequences from L1-Ta family. (**b**) Two sequences from L1-Ta and AluY families.

The 3′UTR regions of L1 transposons are poorly conserved regions that lack sequence similarity even in members of one family. Figure 1 presents dotplots of 50 bp sequences from 3′-ends of two L1s of L1-Ta family and of representatives from L1-Ta and AluY families. However, as we showed earlier, despite the absence of similarity at the sequence level, all L1 and Alu transposons possess a 3′-end stem-loop structure^12^. This observation raises a question about the evolutionary importance of this structure in the process of transposition and whether evolutionary structural constrains exist that can be detected by computational methods.

#### Recognition of 50 bp 3′-end of Alus and L1s

**Figure 2:**
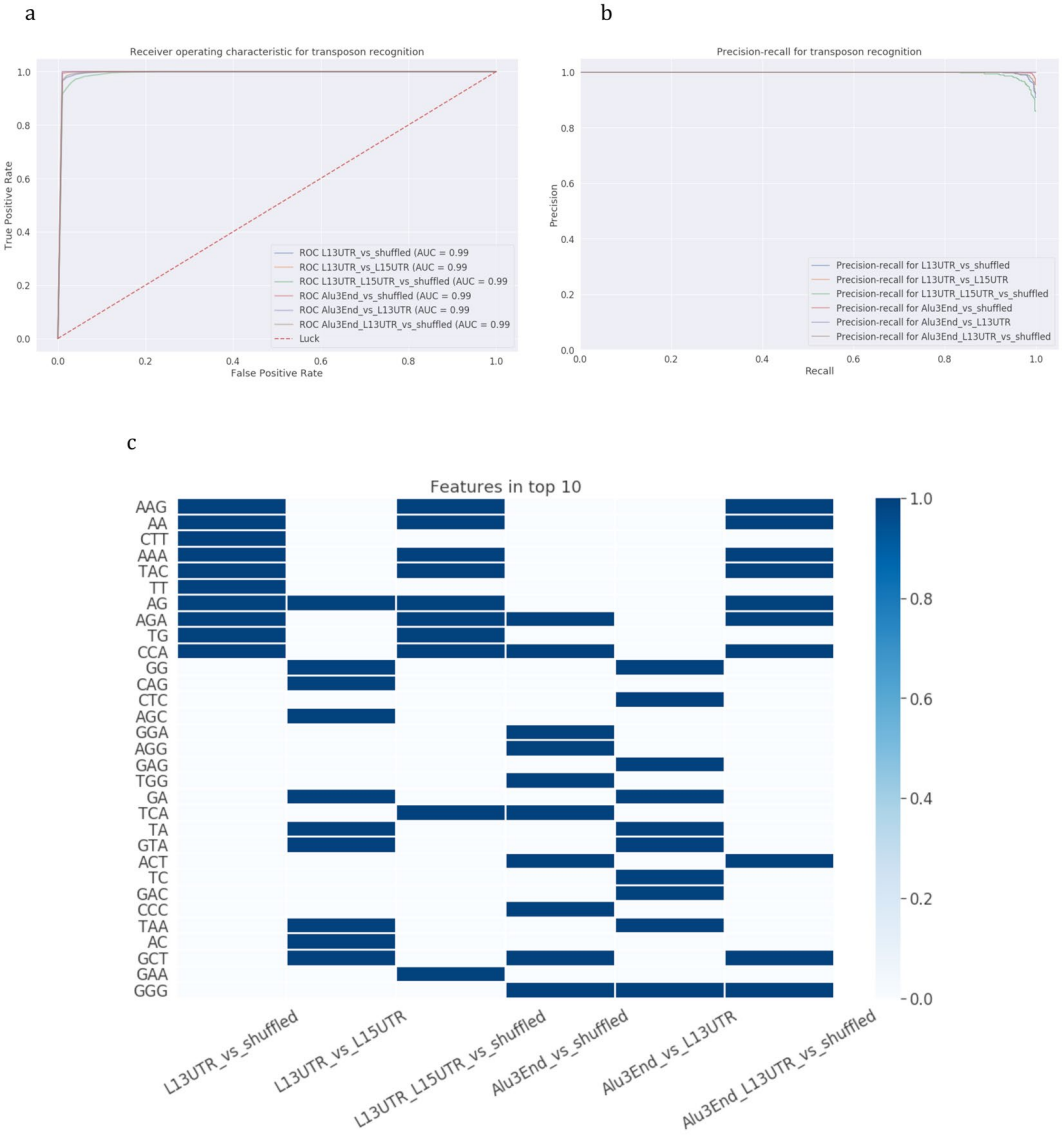
Recognition of 3′-end 50 bp of L1 and Alu elements. Six classification models were constructed: (1) L1 3′UTR vs shuffled, (2) L1 3′UTR vs L1 5′UTR, (3) L1 (L1 3′UTR + L1 5′UTR) vs shuffled, (4) Alu 3′-end vs shuffled, (5) Alu 3′-end vs L1 3′UTR, (6) Alu 3′-end and L1 3′UTR vs shuffled. **(a**,**b)** Models’ performance: **(a)** ROC-curves and **(b)** precision-recall curves. **(c)** Feature importance analysis. Top-10 important parameters are coloured.

Before building the model to recognize specifically 3′-end stem-loops, we first built models trained to recognize the last 50 bp of Alu and L1 sequences when compared to shuffled sequences. We built machine learning models based on sequence composition, taking into account di- and trinucleotide frequencies (see Methods). As an alternative class we shuffled sequences using dinucleotide-shuffling algorithm. We implemented six models comparing either L1 or Alu or their joint set with shuffled sequences, and also tested if L1 and Alu 3′-end or L1 3′UTR and 5′UTR can be distinguished. The results of the modeling are presented in the Table 1 and Fig. 2. All six constructed models achieved high performance with AUC> = 97%. They showed that not only 3′-end sequences of both L1 and Alu transposons could be separated from the other genomic regions, but also L1 3′UTR is distinguishable from 5′UTR.

**Table 1:** Recognition of 3′-ends and 3′-end stem-loops of L1 and Alu sequences.

Class 1	Class 2	AUC			Accuracy			Precision			Recall		
		50 bp	SL1	SL2	50 bp	SL1	SL2	50 bp	SL1	SL2	50 bp	SL1	SL2
L1 3′ UTR	shuffled	0.99	0.97	0.98	0.98	0.93	0.94	0.98	0.96	0.98	0.98	0.90	0.90
L1 3′ UTR	L1 5′ UTR	1.00	0.99	0.99	0.99	0.96	0.98	0.99	0.96	0.98	0.99	0.97	0.98
L1 5′ UTR + L1 3′ UTR	shuffled	0.99	0.98	0.98	0.97	0.93	0.94	0.97	0.96	0.99	0.96	0.90	0.89
Alu 3′-end	shuffled	0.99	0.99	0.99	0.99	0.97	0.98	0.99	0.98	0.99	0.99	0.96	0.97
Alu 3′-end	L1 3′ UTR	0.99	0.99	0.99	0.99	0.99	0.99	0.99	0.99	0.99	0.99	0.99	0.99
Alu 3′-end + L1 3′ UTR	shuffled	0.99	0.98	0.99	0.98	0.95	0.97	0.98	0.97	0.99	0.98	0.93	0.94

Note: 50 bp designates models for recognizing 50 bp 3′-ends, SL1 designates models for recognizing 3′-end stem-loops with sequence-based models, and SL2 designates models for recognizing 3′-end stem-loops with structure-based models.

Feature importance analysis (Fig. 2c) revealed important di- and tri-nucleotides for each model. The analysis revealed that different groups of di- and tri-nucleotides are detected as the most influential in recognition of 3′-end 50 bp L1 from shuffled and Alu from shuffled sequences. The special interest of this study is recognizing the combined set of L1 and Alu 3′-end 50 bp and extraction of common features characteristic to this set. For the case of 3′-end 50 bp sequences, these features are mostly trinucleotides rather than dinucleotides, and mostly GC-rich trinucleotides reflecting the existence of compositional bias of 3′-ends of both transposons.

#### Recognition of 3′-end stem-loops of Alus and L1s with sequence-based models

Next we extracted sequences from 3′-end stem-loops and built two types of models – using only sequence characteristics and only structural, physical and chemical characteristics and position-specific sequence information about loops and bulges (see Methods). As an alternative class we used shuffled sequences.

**Figure 3:**
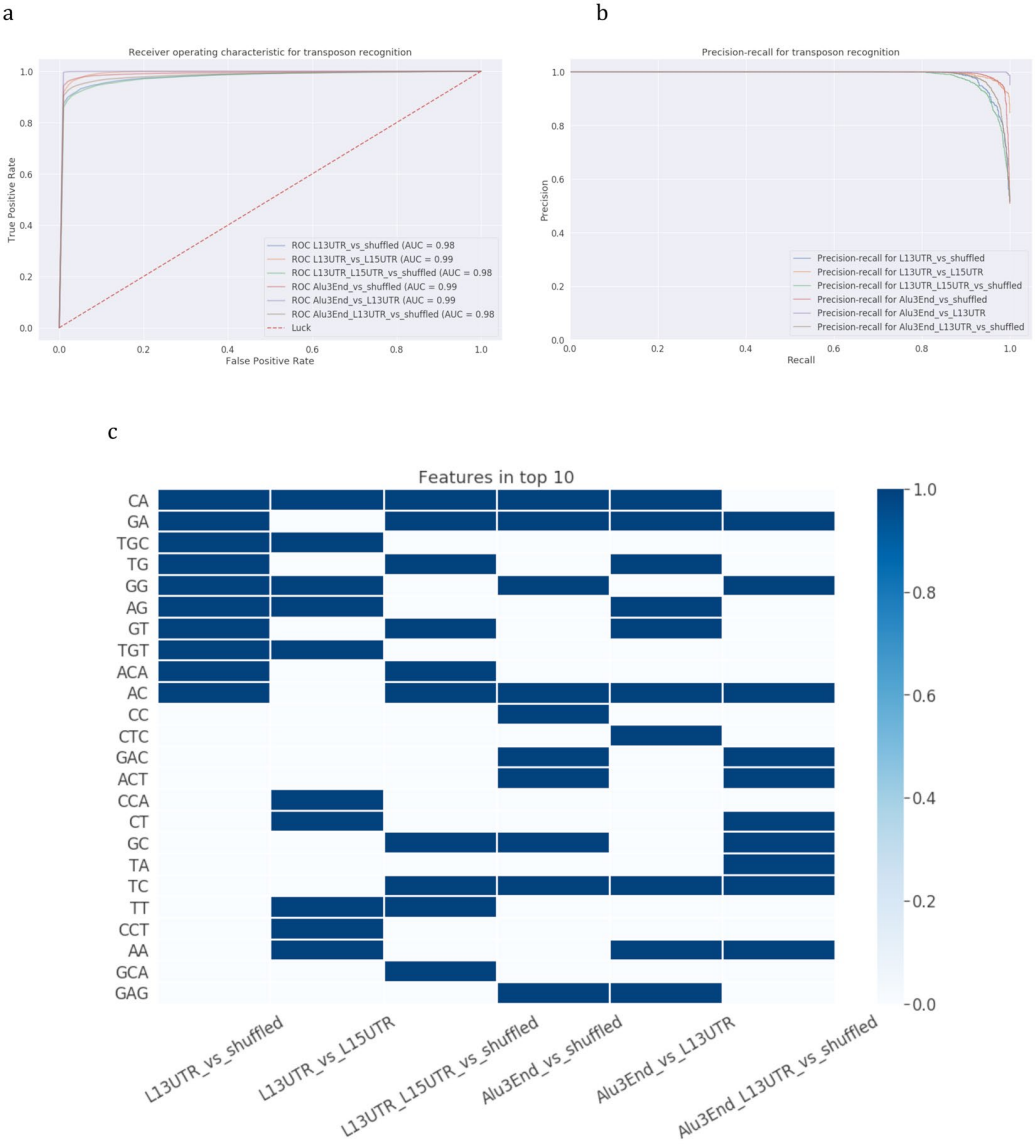
Recognition of 3′-end stem-loops of L1 and Alu elements with sequence-based models. Six classification models were constructed: (1) L1 3′UTR vs shuffled, (2) L1 3′UTR vs L1 5′UTR, (3) L1 (L1 3′UTR + L1 5′UTR) vs shuffled, (4) Alu 3′-end vs shuffled, (5) Alu 3′-end vs L1 3′UTR, (6) Alu 3′-end and L1 3′UTR vs shuffled. **(a**,**b)** Models’ performance: **(a)** ROC-curves and **(b)** precision-recall curves. **(c)** Feature importance analysis. Top-10 important parameters are coloured.

The results of the modeling using only sequence characteristics to recognize 3′-end stem-loops are presented in Table 1 and Fig. 3a,b. In recognizing L1 3′UTR or Alu 3′-end stem-loops or their combined set from stem-loops from shuffled sequences all models achieved performance AUC> = 0.96. In general, sequence-based models for recognition of stem-loops show the same performance as models recognizing 50 bp-ends of transposons, which are the expected results.

Feature importance analyses (Fig. 3c) revealed that top-10 most important features are mostly dinucleotides both for recognizing L1 and Alu 3′-end stem-loops jointly or separately, and these are inverse complementary pairs of dinucleotides such as CA-GT, TC-GA for the experiments L1 *vs* shuffled and AG-CT, TC-GA for Alu *vs* shuffled reflecting inner constraints of the analyzed sequences.

#### Recognition of 3′-end stem-loops of Alus and L1s with structure-based models

Finally, we built machine learning models considering structural and physical properties of a stem and taking into account bulges and loops (see Methods). We considered position-specific dinucleotide properties of a stem including geometrical characteristics such as twist, rise, tilt, bent, shift and slide and also physical and chemical characteristics such as enthalpy, entropy, Gibbs free energy, and hydrophilicity. For loops we considered position-specific sequence composition taking into account only the first five positions of the loop creating 20 binary features describing a loop. For bulges, we considered a bulge size of 3 nucleotides from the left and/or from the right part of a stem. All bulge positions also were fixed and 24 binary features were created to characterize a bulge (see Methods). The results of the modelling are presented in Table 1.

Feature Importance analysis revealed that L1 and Alu 3′end stem-loops are recognized from shuffled stem-loops based on almost different sets of top-10 features, however the analysis of the joint set of L1 and Alu 3′-end stem-loops captured the properties that are important for both transposons.

**Figure 4:**
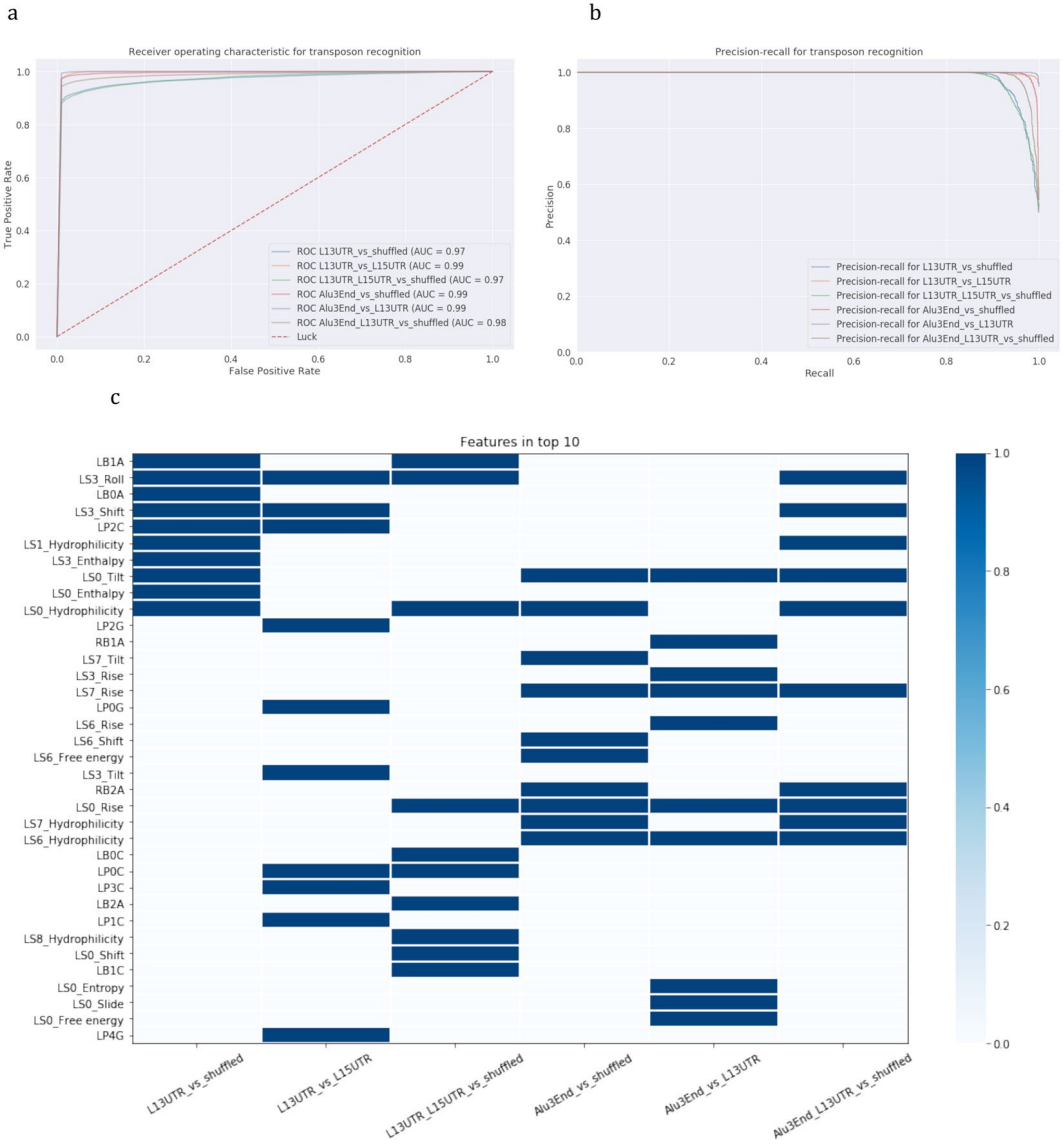
Recognition of 3′-end stem-loops of Alu and L1 elements with structure-based models. (1) L1 3′UTR vs shuffled, (2) L1 3′UTR vs L1 5′UTR, (3) L1 (L1 3′UTR + L1 5′UTR) vs shuffled, (4) Alu 3′-end vs shuffled, (5) Alu 3′-end vs L1 3′UTR, (6) Alu 3′-end and L1 3′UTR vs shuffled. **(a**,**b)** Models’ performance: **(a)** ROC-curves and **(b)** precision-recall curves. **(c)** Feature importance analysis. Top-10 important parameters are coloured.

The structural characteristics shift, roll, and tilt appeared to be the most important parameters in recognizing L1 3′UTR stem-loop from stem-loops from shuffled sequences (shuffled stem-loops) at two stem positions counting from the loop (positions LS0, close to the loop, and LS3, close to the bulge in Fig. 4c). In distinguishing Alu from shuffled stem-loops, many parameters are identified as important for the first stem position counting from the loop (position LS0 in Fig. 4c), and they include energy characteristics such as enthalpy and free energy; geometrical parameters rise and tilt, and also hydrophilicity. For the combined set of L1 and Alu 3′-end stem-loops, the top-10 most important parameters also include the parameter rise for two stem positions, shift, tilt, roll, and hydrophilicity for four stem positions (Fig. 4c).

When looking only at positions having at least one important parameter, the first position was identified as significant for three parameters: hydrophilicity, rise and tilt (position LS0 in Fig. 4c); the fourth position (LS3) showed up for two parameters shift and roll. Four out of ten important features for recognition of a joint set L1-Alu 3′-end stem-loops are hydrophilicity at positions close to the loop (LS0-LS1) and at positions close to the base of the stem (LS6-LS7).

**Figure 5:**
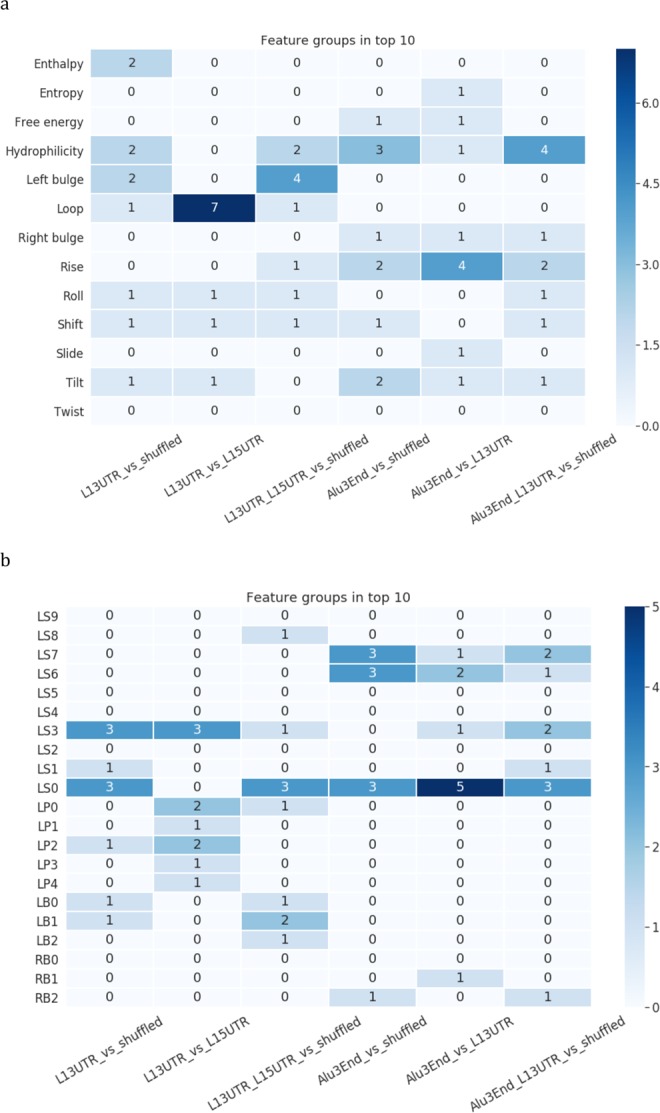
The Top-10 features for recognizing L1 and Alu. (**a**) The top-10 important features grouped by structural and physical parameters characterizing a stem, and parameters related to a loop and a bulge. Numbers in rectangles designate how many times these parameters were selected as important totally in different positions. **(b)** The top-10 important features grouped by positions in a stem (LS0-LS9), loop (LP0-LP4) and bulge (LB0-LB2, RB0-RB2). Numbers in rectangles designate how many times these positions were selected as important totally for different parameters.

The summary of parameters and positions revealed as significant in all six experiments is presented in Fig. 5. The most influential parameter is parameter hydrophilicity (appeared 11 times) followed by the loop-specific positions (9), rise (9), tilt (6), and shift (5) (Fig. 5a). Three stem positions were highlighted as important: first dinucleotide position adjacent to the loop (LS0, 17 times), position close to a bulge (LS3, 10 times), and positions close to a stem base LS6-LS7 (7 times) (Fig. 5b).

### Recognition of 3′-ends and 3′-end stem-loops of processed pseudogenes and mRNAs

Processed pseudogenes are gene copies that result from the process of mRNA retrotransposition. We examined processed pseudogenes and mRNAs for the presence of stem-loops at their 3′-ends (see Methods). The human genome contains around ten thousand processed pseudogenes. We found that 62% (6,309 out of 10,144) of all processed pseudogenes have stem-loops at the 50 bp 3′-ends. Distribution of the number of copies of processed pseudogenes in the human genome follows the power-law distribution, when few genes have multiple copies and many genes only few^25^. Ribosomal proteins account for 22% of all processed pseudogenes, that is why we additionally checked for ribosomal proteins and found that around 70% of those have stem-loops at their 3′-end (194 out 281). We also examined all cellular mRNAs for the presence of stem-loop structures, and found that 64% of all mRNAs (53,094 out of 82,960) have stem-loops at the 50 bp 3′-ends. When counting only protein-coding mRNAs, their percentage increased up to 68% (20,328 out of 29,636).

**Figure 6:**
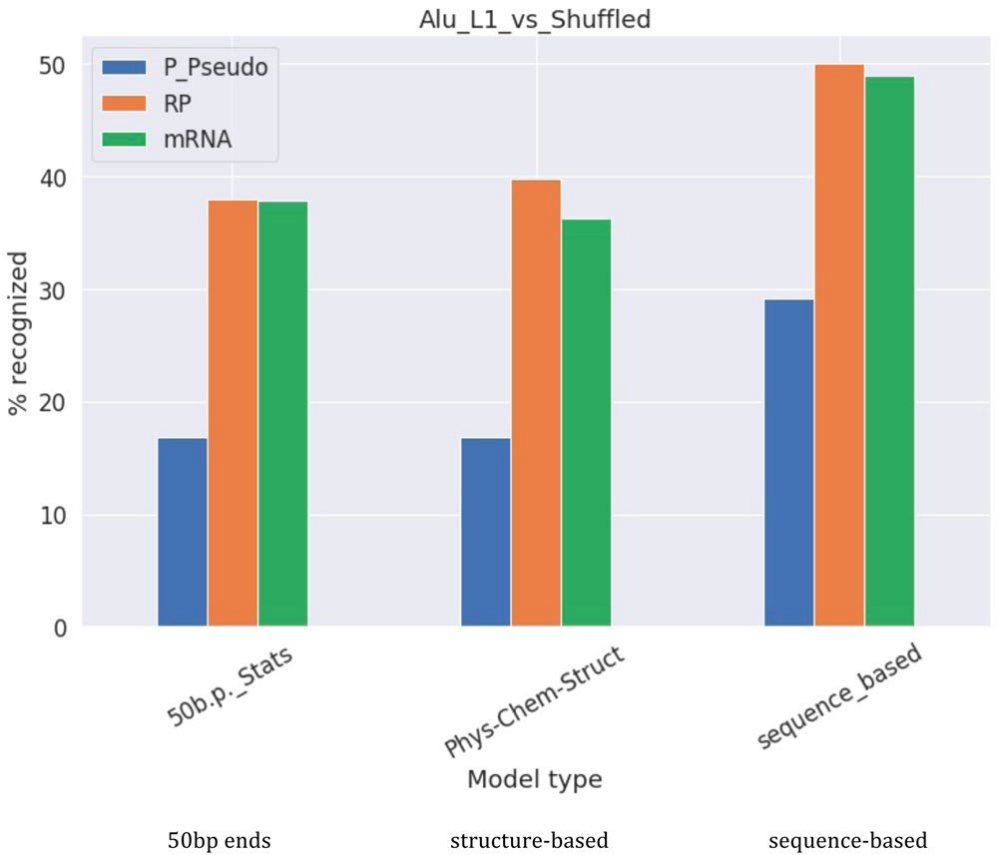
Percentage of processed pseudogenes, ribosomal proteins and mRNAs recognized by models trained to recognize L1 3′-ends or 3′-end stem-loops.

L1-recognizing model incorporates properties inherent only to L1, and Alu-recognizing model incorporates properties inherent only to Alu; it is evident from Figs 2–4 that they do not overlap. The joint Alu + L1 model incorporates properties inherent to both transposons and we further checked whether the models, trained to recognize the joint set of L1 and Alu 3′-ends or 3′-end stem-loops, are able to recognize 3′-ends or 3′-end stem-loops of processed pseudogenes, ribosomal proteins and mRNAs (Fig. 6). It appears that the model, trained to recognize L1-Alu 50 bp 3′-ends, recognizes around 40% of ribosomal proteins and mRNAs and only 17% of processed pseudogenes. However, models trained to recognize L1-Alu 3′-end stem-loops could recognize around 50% of stem-loops of mRNAs and ribosomal proteins, and around 30% of processed pseudogenes, if using the sequence-based model; the structure-based model behaves as the 50 bp-end model. A low percentage of recognition of processed pseudogenes is explained by the fact that the processed pseudogenes are “dead on arrival” and quickly accumulate mutations; some are even 3′-end truncated. What we can conclude from this experiment is that at least half of mRNA has common recognizable properties with the joint L1-Alu set, and these properties are shared for both 3′-ends and for 3′-end stem-loop structures.

**Figure 7:**
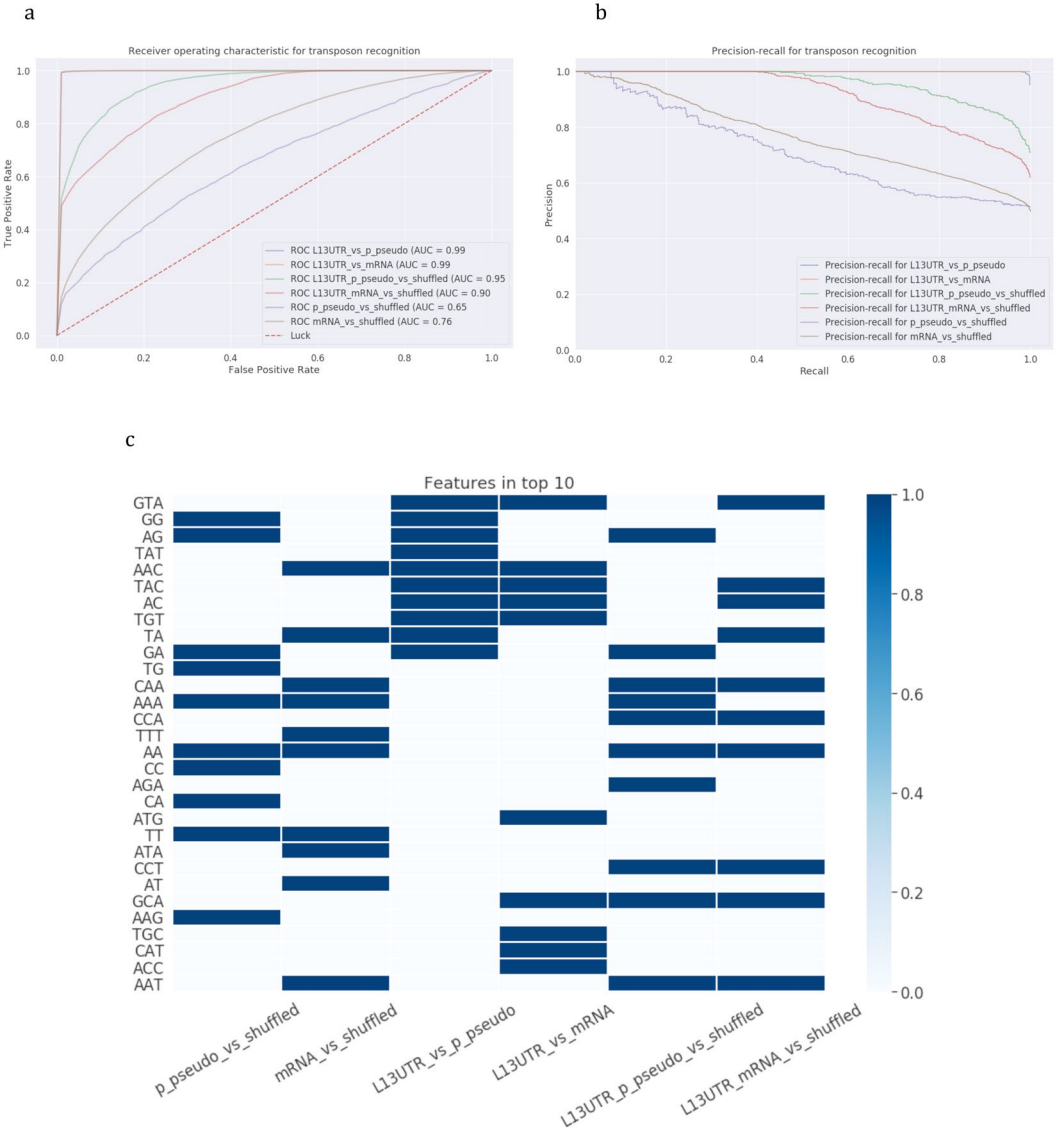
Recognition of 3′-end 50 bp of processed pseudogenes and mRNAs. Six classification models were constructed: (1) processed pseudogenes vs shuffled, (2) mRNAs vs shuffled, (3) L1 3′UTR vs processed pseudogenes, (4) L1 3′UTR vs mRNAs, (5) L1 3′UTR and processed pseudogenes vs shuffled, (6) L1 3′UTR and mRNAs vs shuffled. **(a,b)** Models’ performance: **(a)** ROC-curves and **(b)** precision-recall curves. **(c)** Feature importance analysis. Top-10 important parameters are coloured.

**Figure 8:**
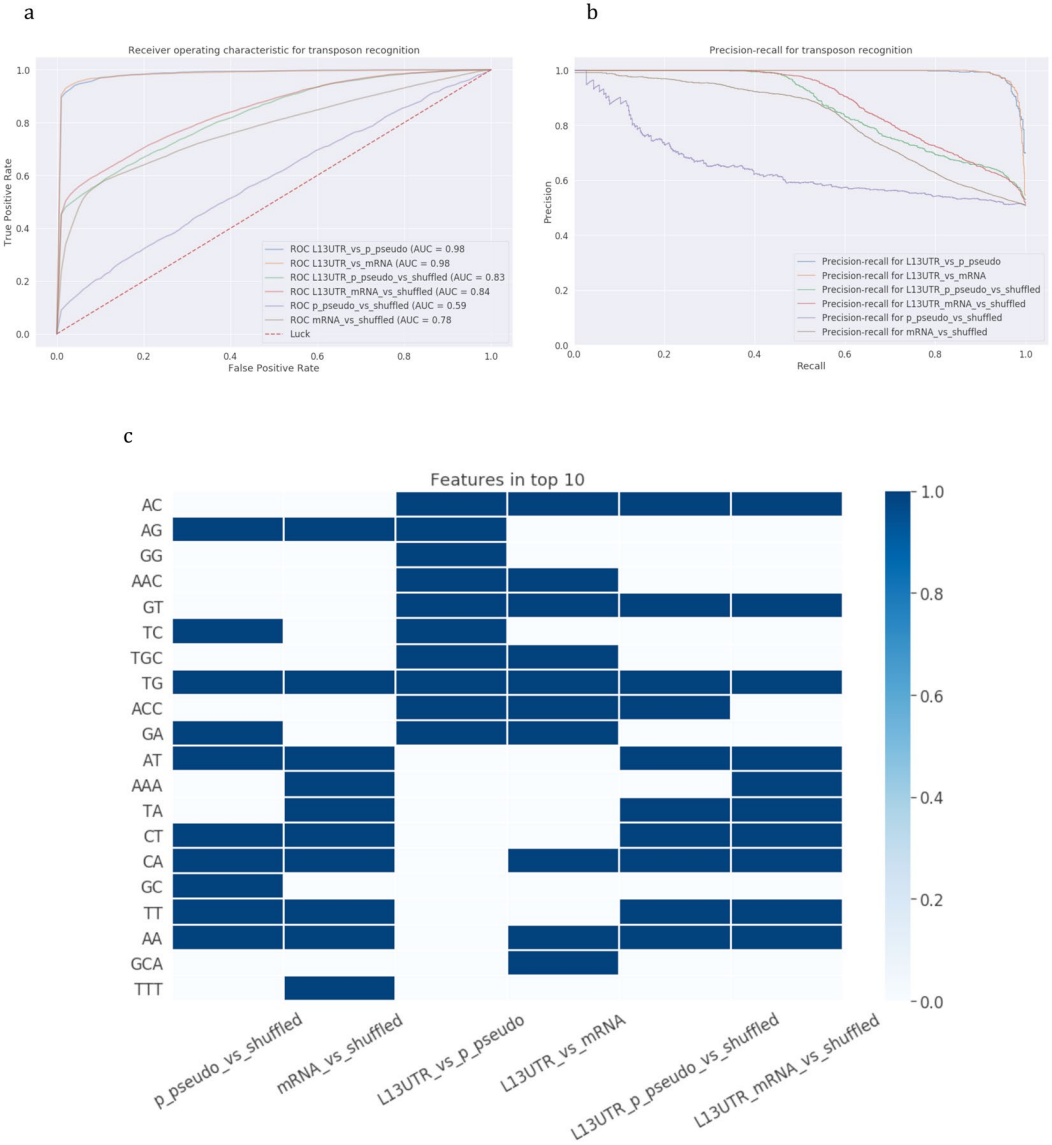
Recognition of 3′-end stem-loops of of processed pseudogenes and mRNAs with sequence-based models. Six classification models were constructed: (1) processed pseudogenes vs shuffled, (2) mRNAs vs shuffled, (3) L1 3′UTR vs processed pseudogenes, (4) L1 3′UTR vs mRNAs, (5) L1 3′UTR and processed pseudogenes vs shuffled, (6) L1 3′UTR and mRNAs vs shuffled. **(a**,**b)** Models’ performance: **(a)** ROC-curves and **(b)** precision-recall curves. **(c)** Feature importance analysis. Top-10 important parameters are coloured.

**Figure 9:**
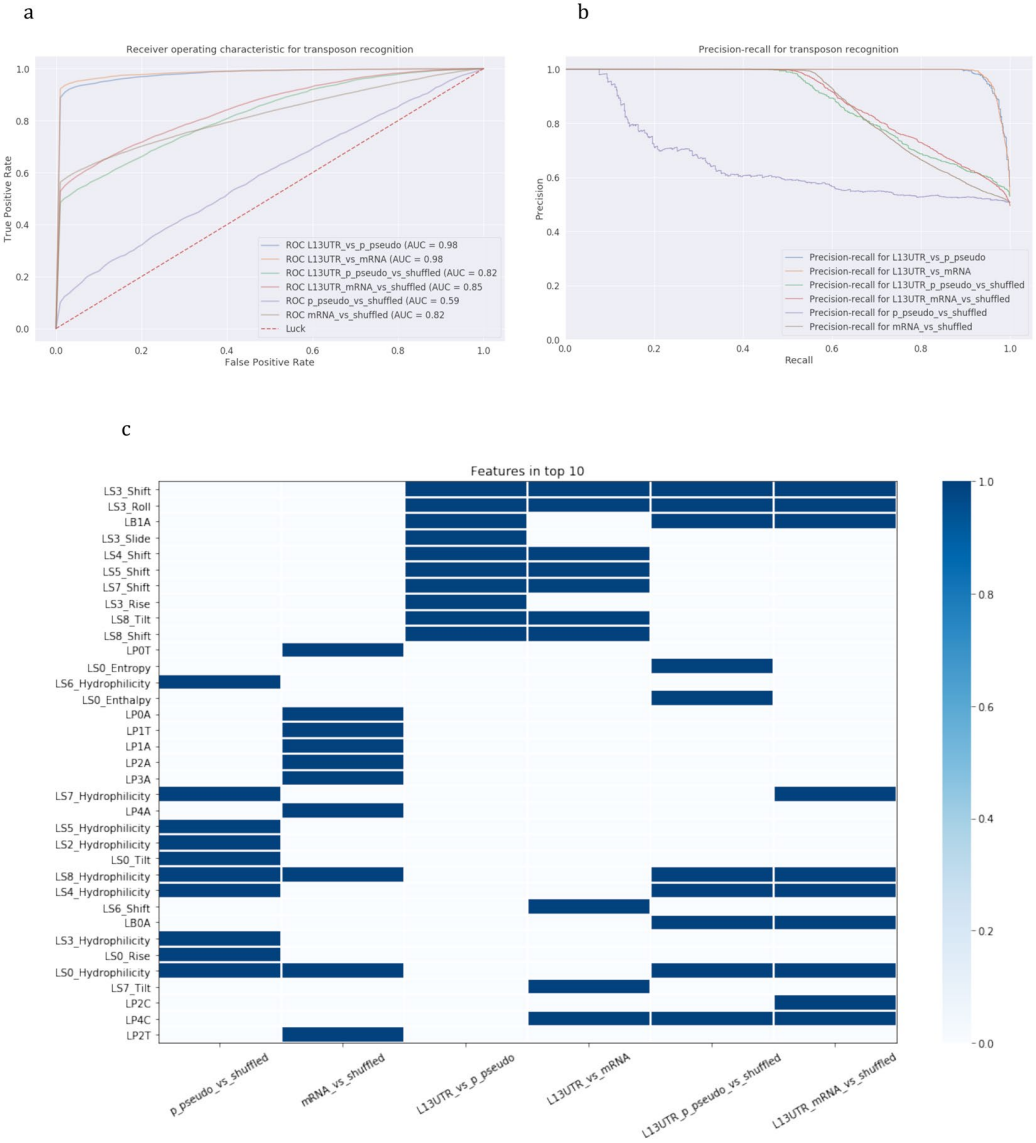
Recognition of 3′-end stem-loops of processed pseudogenes and mRNAs with structure-based models. Six classification models were constructed: (1) processed pseudogenes vs shuffled, (2) mRNAs vs shuffled, (3) L1 3′UTR vs processed pseudogenes, (4) L1 3′UTR vs mRNAs, (5) L1 3′UTR and processed pseudogenes vs shuffled, (6) L1 3′UTR and mRNAs vs shuffled. **(a**,**b)** Models’ performance: **(a)** ROC-curves and **(b)** precision-recall curves. **(c)** Feature importance analysis. Top-10 important parameters are coloured.

To obtain information about specific common properties of L1 and mRNA/processed pseudogenes, we repeated the same set of experiments, i.e. those performed for L1s and Alus, for processed pseudogenes and mRNAs. We built models to recognize 50 bp 3′-ends and 3′-end stem-loops of processed pseudogenes and mRNAs together with L1s or separately and extracted the most important parameters of the models. The modeling results are presented in Table 2 and in Figs 7–9.

**Table 2:** Recognition of 3′-ends and 3′-end stem-loops of mRNAs and processed pseudogenes.

Class 1	Class 2	AUC			Accuracy			Precision			Recall		
		50 bp	SL1	SL2	50 bp	SL1	SL2	50 bp	SL1	SL2	50 bp	SL1	SL2
processed pseudo-genes	shuffled	0.65	0.59	0.59	0.61	0.56	0.56	0.62	0.57	0.56	0.58	0.55	0.54
mRNA	shuffled	0.76	0.78	0.82	0.68	0.71	0.77	0.68	0.73	0.83	0.69	0.68	0.66
L1 3′ UTR	processed pseudo-genes	0.99	0.99	0.98	0.99	0.95	0.94	0.99	0.96	0.98	0.98	0.93	0.91
L1 3′ UTR	mRNAs	0.99	0.99	0.99	0.99	0.96	0.96	0.99	0.97	0.98	0.94	0.94	0.93
L1 3′ UTR + processed pseudo-genes	shuffled	0.95	0.83	0.83	0.87	0.73	0.74	0.85	0.76	0.80	0.90	0.68	0.63
L1 3′ UTR + mRNA	shuffled	0.90	0.85	0.99	0.80	0.75	0.96	0.81	0.78	0.98	0.78	0.70	0.93

Note: 50 bp designates models for recognizing 50 bp 3′-ends, SL1 designates models for recognizing 3′-end stem-loops with sequence-based models, and SL2 designates models for recognizing 3′-end stem-loops with structure-based models.

In the case of processed pseudogenes and mRNAs we do not observe the same high performance as that of the models built using the same approach for Alus. Characteristically, models trained to recognize 3′-ends or 3′-end stem-loops of processed pseudogenes only have the worst performance: AUC of 0.65 in recognizing 50 bp 3′-ends and AUC of 0.59 for recognizing 3′-end stem-loops. This can be explained by the fast rate of accumulating mutations by the processed pseudogenes. In comparison, 3′-ends of mRNAs can be distinguished with a higher performance (AUC = 0.76), and mRNA 3′-end stem-loops are recognizable even better with structure-based models (AUC = 0.82). The combined set of 50 bp 3′-ends from L1s and processed pseudogenes can be distinguished from shuffled sequences with a high performance (AUC = 0.95). Feature importance analysis revealed (Fig. 7c) that the important parameters are mostly AT-rich di- and trinucleotides, and almost the same set of di- and trinucleotides are identified as significant in the joint set of mRNAs and L1s.

The analysis of 3′-end stem-loops showed that important features are grouped in pairs of processed pseudogenes and mRNAs, both in sequence-based and structure-based models (Figs 8c and 9c): either processed pseudogenes and mRNAs are compared to shuffled sequences, L1s, or they are combined in a joint set with L1s. In distinguishing joint sets of mRNAs and L1s the structure-based models showed very high performance (AUC = 0.99) compared to sequence-based models (AUC = 0.85). The difference in the recall is considerably higher (0.93 vs 0.75, see Table 2), indicating that the model is well trained to specifically recognize stem-loops of interest. The most important features in recognizing either joint set of L1s- mRNAs or L1s-processed pseudogenes were the shift, roll, hydrophilicity, which appeared to be the same salient features in models for recognizing the joint L1 and Alu sets. Processed pseudogenes have additionally entropy and enthalpy parameters included in the top-10 features, and mRNAs have four loop and bulge positions.

**Figure 10:**
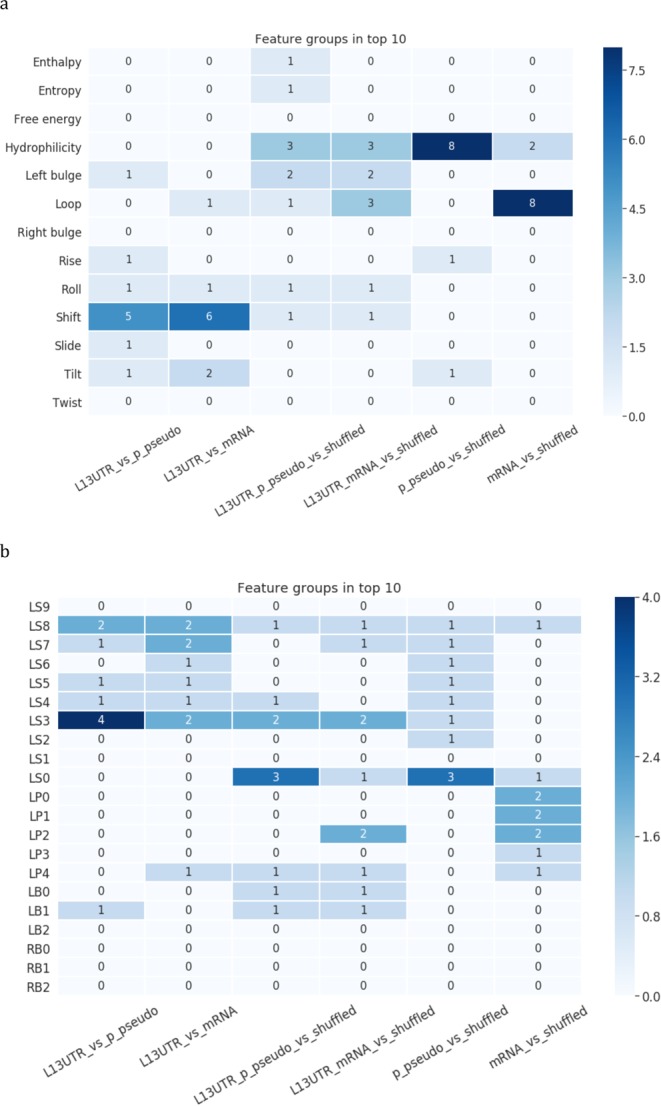
The Top-10 features from 6 models for recognizing L1 and processed pseudogenes or mRNAs. (**a**) The top-10 important features grouped by structural and physical parameters characterizing a stem, and parameters related to a loop and a bulge. Numbers in rectangles designate how many times these parameters were selected as important totally in different positions. **(b)** The top-10 important features grouped by positions in a stem (LS0-LS9), loop (LP0-LP4) and bulge (LB0-LB2, RB0-RB2). Numbers in rectangles designate how many times these positions were selected as important totally for different parameters.

The summary of parameters and positions identified as significant in all experiments with processed pseudogenes and mRNAs is presented in Fig. 10. The most influential parameter is hydrophilicity (showed 16 times), followed by shift (13) and the loop-specific positions (13) (Fig. 10a). Three stem positions were highlighted as important: position close to a bulge (LS3, 11 times), position close to a loop (LS0, 8 times), and position close to a stem base LS8 (8 times) (Fig. 10b). The same parameters and positions, though in a different order, were identified in the models of L1-Alu recognition (Fig. 5).

## Discussion

In the present study, we explored the ability of machine-learning models to recognize 3′-ends and 3′-end stem-loops of active transposons in human genome: L1 and Alu separately and as a combined set as well as L1 and Alu or L1 5′UTR and 3′UTR between each other. We evaluated two types of machine-learning models using different types of features, encoding sequence composition and physical, chemical, and geometrical properties of dinucleotides, calculated from different experiments. Sequence-based models use nucleotide composition of an entire sequence, while structure-based models take into account structural properties of a stem and position-specific properties of loops and bulges.

Here there is a need to clarify that, in essence, the so-called structure-based model also uses sequence information but is mapped to a different alphabet of dinucleotide characteristics. Even though all these characteristics have been obtained from experiments, the experiments were done for a narrow class of RNA duplexes. Thus, to obtain structural properties of RNA dinucleotides X-ray crystallographic images of RNA duplexes were analyzed and later filtered for structures containing proteins, drugs, mismatches, overhanging bases or unusual bases in canonical Watson–Crick pairings^26^. The authors performed helical analysis of the remaining structures at the canonical base-pair level. The structures that showed deviations (more than three standard deviations) of helical parameters were also removed from the analyzed dataset^26^. As a result, the resulting translational (shift, slide and rise) and rotational (tilt, roll and twist) parameters were shown to correspond to averages for crystallographic static images of naked RNA. As was stated in^26^ a sizeable amount of experimental data was removed due to filtering, and it guarantees that perturbations were within the harmonic limit. Also, the filtering removed major part of AT dinucleotides, whose presence corresponds to flexibility. Entropy, enthalpy and free energy used in this study were taken from^27^, and these parameters are derived from optical melting of RNA duplexes taking into account compositions of ends. Hydrophilicity for 16 dinucleotides were also calculated from experiments as described in^28^.

All of the selected features correspond to dinucleotides and, in fact, in the model we use numerical representations of the sequence-based information. However, this approach has two advantages. First, machine-learning algorithms work better with numerical values rather than with binary one-hot encoding or integers resulting from counts of k-mers. Secondly, they can capture some tendencies related to dinucleotides and thus can be interpreted.

In a set of L1-Alu experiments, both models showed comparable performance (AUC > 96–99%), however feature importance analysis of the structure-based models allowed revealing properties that are more influential for L1 and Alu 3′-end stem-loops. Among these properties are properties shift, rise, tilt, and hydrophilicity.

We also examined processed pseudogenes and cellular mRNAs for the presence of stem-loop structures at their 3′-ends and constructed the same sets of machine learning models as those for Alus. Those models that were trained to detect 3′-ends or 3′-end stem-loops in processed pseudogenes, achieved performance no higher than 0.65, which can be explained by the fact that the processed pseudogenes are considered “dead on arrival”, and accumulate mutations without any selection constrains. The performance of other models ranges from 0.76 for recognizing 50 bp mRNAs to 0.99 for recognizing 3′-end stem-loops of the joint set of L1s and mRNAs with structure-based models, or for distinguishing between L1 3′-end stem-loops from those of mRNA or of processed pseudogenes. Three important parameters in recognizing joint sets of L1s and pseudogenes or mRNAs are similar to those in recognizing joint sets of L1s and Alus: shift, roll, hydrophilicity and specific loop positions. Also the same specific stem positions showed as significant in all sets of experiments, either for L1-Alu, L1-mRNA, or L1-processed pseudogenes: positions close to a bulge, positions close to a loop, and positions close to the stem base.

The importance of stem-loop structural parameters was shown earlier in a series of experiments on transposons of different species. Experimental work on solution structures of the 3′-end stem-loop in eel transposons revealed that the sequence GGRNA of the loop forms the structure that is recognized by UnaL2 RT^13^. One of the special characteristics of this structure is that the fourth residue, uracil in the case of the investigated loop, is exposed to the solvent, however, its replacement does not affect retrotransposition. However, mutation of the second residue, guanine, abolished the retrotransposition activity. This led to the conclusion that this residue is required for a specific recognition of the UnaL2 RT. The deletion of the fourth residue results in the sequence of GGAA, which becomes a well-known tetraloop GNRA^29^. Solution structures of three tetraloops GAGA, GCAA and GAAA revealed that the three last bases are usually stacked and the first base is located on the other side from three stacked bases. This arrangement creates a sharp turn between the first and the second nucleotides resembling U-turn^30^, known as a motif for tertiary interactions.

In zebrafish, 3′-end stem-loops in two transposons ZfL2-1 and ZfL2-2 were investigated which part of stem-loops are important for retrotransposition^9^. Two stem-loops differ from each other by ZfL2-1 having an insertion in the form of another stem-loop in the position of the fourth residue of the loop. First, RT transposes only cognate stem-loop. Experiments with changing loops and stems creating hybrid stem-loops composed of different stems and loops revealed that replacement of a loop and leaving cognate stem leads to complete abolishment of retrotransposition while leaving cognate loop and replacing stem reduced transposition rate only by half. This suggests the loop is important for RT specific recognition.

The crystal structure of NHL, RNA-binding domain, from fly Brat protein in complex with RNA showed an interaction, where a guanine base is turned in the opposite direction compared to other motif bases, and it protrudes into a binding pocket^31^. The crystal structure of NHL domain from worm LIN-41 protein revealed^32^ that domain binds to stem-loop which shape determines the binding specificity. This stem-loop consists of three bases and all three loop nucleotides −1 and +1 bases of the stem are participating in specific binding. Thus, NHL domain in the fly and in the worm has different binding mechanisms suggesting high evolutionary plasticity.

The crystal structure of stem-loop binding proteins in complex with stem-loop and 3′hExo revealed that the entire stem-loop and its flanking sequences participate in binding with two proteins^33^. It was shown that the proteins recognize the shape of the stem-loop rather than sequence however sequence determines the shape. The specificity of recognition is provided by the nucleotides in the loop: the first (U) and the third (U) nucleotides are highly conserved.

Here, we probed RNA structural parameters characteristic for stem-loops at the 3′-end of L1 and Alu sequences, as well as at the 3′-ends of processed pseudogenes and mRNAs, with machine learning models. The results of the modeling revealed both stem and loop parameters among the top 10 most important features either in recognizing only L1 and only Alu 3′-end stem-loops or a combined set of Alu and L1 elements. The same is true for a joint set of L1s and processed pseudogenes or mRNAs, while in recognizing only processed pseduogenes or only mRNAs most important parameters include only hydrophilicity and loop positions.

**Figure 11:**
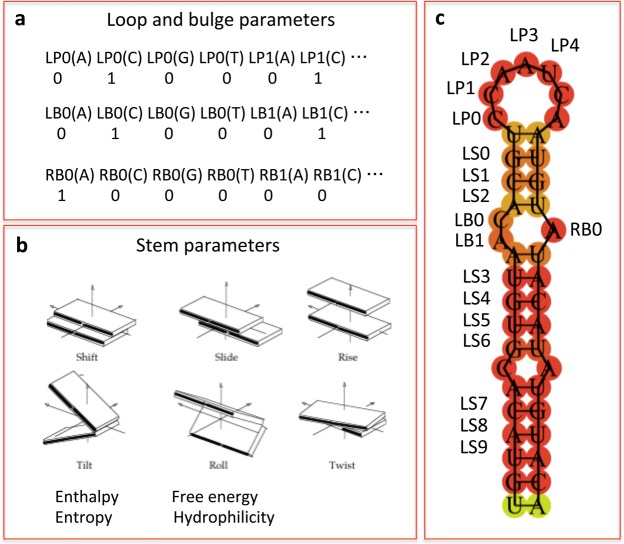
Creating property vector based on RNA structural properties (see Methods for details). **(a)**. Binary property vector for a loop and a bulge. **(b)**. Structural, physical and chemical parameters for a stem. (**c**). Stem-loop with designated positions for a stem, loop and bulge.

The parameter shift as well as rise and slide are translational helical parameters as opposed to rotational (tilt, roll and twist)^26^. Shift reflects the translation around X-axis, slide – translation around Y-axis and rise – translation around Z-axis (Fig. 11b). They influence the height and width of the double-stranded RNA that would be reflected in the height and width of the stem. All of them contribute to the flexibility of A-RNA, which in case of transposon stem-loops appeared to be a subject for evolutionary selection. The importance of the energy parameters of the dinucleotide adjacent to the loop as in the case of Alu stem-loops reflects structural peculiarity of Alu families since the energetic parameters did not appeared as influential in L1 stem-loops. Cellular mRNA is a class of very diverse objects consisting of protein-encoding RNA, tRNAs, rRNAs and various classes of regulatory RNAs. This explains why the models could not capture any regularities for dinucleotide properties at specific positions of stems. Instead the most important parameters are either hydrophilicity or loop positions.

The conducted experiments cannot not fully answer the question about the role of stem-loop in pseudogenization of mRNA, but they assert such a role exists: first, the 3′-end stem-loop is a property of around 70% of mRNA; secondly, around 50% of mRNA can be recognized by a model based on the common properties of either 50 bp 3′-end or 3′-end stem-loop of the joint L1-Alu set; thirdly, stem-loop structures are recognized better than just 50 bp-ends; fourth, the joint set of L1 and mRNA is recognized with a good performance (AUC > 0.9) reflecting the existence of common properties for that set. The set of ribosomal proteins that account for 22% of all processed pseudogenes percentagewise are recognized no better than mRNA. This probably reflects randomness in selection of those genes that become retropseudogenes.

The trained models have limitations: feature set is preselected and might not be the optimal. As of today there does not exist a standard machine learning approach to predict RNA (DNA) secondary structures. Different machine learning methods have their advantages and disadvantages. For example, quadruplexes were predicted with high performance with eXtreem Gradient Boosting method^22^ based on sequence composition only (k-mers frequencies), using experimental data from G4-seq for training^34^. Lately, CNN has shown to be slightly in advance as compared to other machine-learning approaches. The advantage of CNN is that there is no need to select features for input data, they are extracted automatically from an input sequence. Usually an input for CNN is a DNA sequence transformed with one-hot encoding to binary matrix. Several studies incorporated RNA structural information into sequence data^35,36^. In recognizing non-coding RNA with CNN, the input data also included probabilities of a nucleotide being either paired or non-paired^35^. When predicting RNA-protein binding the probabilities were ascribed to five states: a nucleotide in the hairpin loop, inner loop, multi loop, external region, or in the paired state^36^. In both methods, adding structural information improved CNN performance. However the disadvantage of neural networks is difficulty of interpretation. Some information can be retrieved from CNN through selected filters, but this information is actually sequence logos of common motifs; no information about underlying structures or structural motifs has been obtained so far from CNN filters.

Our approach is structure-centric, based on feature selection and thus having an advantage in interpretation. With representation of RNA structures through averaged properties of dinucleotides and loop and bulge composition, one can capture predominate properties of the analyzed structures – whether geometrical properties or else energy characteristics, or chemical, or loop-specific features are more influential. In our task geometrical properties appeared to be more important for evolutionary young sequences (such as full length copies of L1 and Alu), while when comparing the entire range of mRNA or pseudogenes neither structural nor energy features were selected; the selected features included only hydrophilicity and loop positions, reflecting the heterogeneity of objects.

The proposed approach is useful in analysis of RNA (DNA) secondary structures for recognizing different classes of non-coding RNA, and, in general, of functional stem-loops playing important roles as promoters, attenuators, splicing, intermolecular interactions (triplex structures), and others. The method allows detecting different structural constrains inherent to different classes of the analyzed structures.

## Conclusion

We constructed machine learning models that are able to recognize 3′-end L1 and Alu stem-loops, and 3′-ends of processed pseudogenes and mRNAs, based on different types of information. The models of the first type use only information on sequence composition, while models of the second type use physical, chemical and geometrical properties of RNA. We applied these models to a series of experiments on recognizing 50 bp 3′-ends and 3′-end stem-loops of L1s, Alus, processed pseudogenes and mRNAs both jointly and separately. In case of L1-Alu experiments, both types of models showed high performance with AUC > 0.96–98; for experiments with L1-processed pseudogenes and L1-mRNAs, the model performance is lower with AUC in the range of 0.83–0.99.

The feature importance analysis of the structure-based model revealed structurally important characteristics in stem-loop structures of different groups of elements. Thus, the parameters shift, rise and tilt appeared to be more important for recognizing the joint set of L1 and Alu stem-loops, while shift was shown to be more important for recognizing L1 3′-UTR stem-loops and rise was more important for distinguishing between L1 and Alu 3′-end stem-loops. Additionally, the parameters of stem positions adjacent to the loop appeared to be more important for Alu recognition. For recognizing 3′-end stem-loops of mRNAs and pseudogenes, the most important parameters were hydrophilicity and AT-rich loop positions.

The obtained results clearly point to the existence of structural constrains for 3′-end stem-loops of L1 and Alu, which presumably play an important role in recognition of L1-Alu pairs by the L1 machinery. They also highlight the potential of mRNAs to be recognized and retrotransposed by L1 machinery. The constructed machine-learning models are not restricted to recognition of transposon-related stem-loops, but have a broader range of applications in recognizing different classes of functional stem-loops and can be scaled to the task of predicting RNA (DNA) secondary structures with machine-learning methods.

## Methods

### Sets of L1 and Alu transposons

Full-length L1 transposons were taken from an evolutionary study of Khan *et al*.^23^ and it comprises 6622 elements. The selection was done by searching the RepeatMasker annotation of human genome with L1 elements longer than 6 KB.

Full-length Alu transposons were taken from Price *et al*.^24^. AluJ is inactive family and was not taken into consideration^37^. We considered a set of 12,431 AluS (724) and AluY (11,707) sequences (Supplementary Table 4 in Price *et al*.^24^).

L1 and Alu sequences used in this study are available at OSF Data repository: 10.17605/OSF.IO/PZKVN.

### Sets of processed pseudogenes and mRNAs

Sequences for processed pseudogenes were obtained from UCSC genome browser, track GENCODE v28lift37, table wgEncodeGencodePseudoGeneV28lift37 linked to wgEncodeGencodeAttrsV28lift37 with the field geneType containing information on processed pseudogenes. mRNA was downloaded as sequences directly from UCSC genome browser, track UCSC genes, table knownGenes. Protein coding sequences were download from track UCSC genes, table knownCanonical, which contains canonical splice variant of a gene.

Sequences of processed pseudogenes and mRNAs used in this study are available at OSF Data repository: 10.17605/OSF.IO/PZKVN.

### Shuffled sequences

As an alternative class we used shuffled sequences that were obtained with the dinucleotide shuffling method that preserved dinucleotide frequencies. It prevents the models from relying on low-level statistics of genomic regions^38^. We took implementation from https://github.com/wassermanlab/BiasAway/blob/master/altschulEriksonDinuclShuffle.py and updated it with a newer python version: https://github.com/AlexShein/transposons/blob/master/src/py_scripts/altschulEriksonDinuclShuffle.py.

### Stem-loop annotations

Transposon annotation of sequences with stem-loop structures was performed with the program “DNA Punctuation” (www.dnapunctuation.org) with an open source-code (https://github.com/mariapoptsova/dnapunctuation. The following parameters were used: the stem range of 10–20 bp, the maximum loop length of 10 bp, and the number of mismatches allowed in the stem is 5 bp. For the 3′-end set of stem-loops, we took stem-loops located within the last 50 bp of a transposon sequence, either L1 or Alu. For the 5′-end set of stem-loops, we took stem-loops located within the first 50 bp of transposon sequences. All transposon annotations with stem-loops are available at OSF Data repository: Project “ Recognition of 3′-end L1 and Alu stem-loops in human genome using sequence-based and structure-based machine-learning models” 10.17605/OSF.IO/PZKVN.

We also annotated shuffled sequences with stem-loop structures.

### Feature vectors for sequence-based and structure-based models

For sequence-based model, we took frequencies of di- and trinucleotides counting occurrences of each k-mer moving with the 1 bp step along the sequence.

For structure-based models, we considered a stem, a loop, and a bulge. For stem we took RNA dinucleotide properties from DiPRoDb^16^, which include structural parameters shift, slide, rise, tilt, roll, twist and physical and chemical properties such as enthalpy, entropy, free energy, and hydrophilicity.

We took first the 10 bp of a stem counting from the loop and split it into 9 dinucleotides (Fig. 11). Each dinucleotide was characterized by 10 parameters: 6 structural and 4 physical and chemical. We considered the minimum loop length of 5 bp and created position-specific nucleotide binary vector where each loop position was represented by 4 nucleotides (Fig. 11a). Thus, the loop was represented by 20-dimenisional binary vector. We considered a bulge of a size of 3 bp on each stem thus making 6 bulge positions: 3 on the left stem and 3 on the right stem. Similar to a loop property vector, we characterized each position as a binary vector of 4 nucleotides thus making a 24-dimensional binary vector for bulge positions. If a bulge in a stem-loop structure was less than 3 nucleotides we filled missing positions with zeros. The resulting property vectors consisted of 90 + 20 + 24 = 134 parameters.

Feature vectors compositions are available at github: https://github.com/AlexShein/transposons/.

### Machine-learning models

We built Random Forest models with 2000 trees using scikit-learn library. The model implementation and all data analysis is available at github: https://github.com/AlexShein/transposons/.

## Data Availability

The model implementations and all data analysis are available at github: https://github.com/AlexShein/transposons/. Transposon sequences of L1s and Alus, processed pseudogenes and mRNAs, annotations with stem-loops, and all the data used in this study are available at OSF Data repository: 10.17605/OSF.IO/PZKVN.
